# Caveolin-1 Deficiency Protects Mice Against Carbon Tetrachloride-Induced Acute Liver Injury Through Regulating Polarization of Hepatic Macrophages

**DOI:** 10.3389/fimmu.2021.713808

**Published:** 2021-08-09

**Authors:** Ziheng Yang, Jie Zhang, Yan Wang, Jing Lu, Quan Sun

**Affiliations:** ^1^Department of Laboratory Animal Science, School of Basic Medical Sciences, Capital Medical University, Beijing, China; ^2^Laboratory Animal Center, Capital Medical University, Beijing, China; ^3^Department of Neurology, Xuanwu Hospital, Capital Medical University, Beijing, China

**Keywords:** caveolin-1, hepatic macrophages, macrophage polarization, carbon tetrachloride, acute liver injury

## Abstract

Polarization of hepatic macrophages plays a crucial role in the injury and repair processes of acute and chronic liver diseases. However, the underlying molecular mechanisms remain elusive. Caveolin-1 (Cav1) is the structural protein of caveolae, the invaginations of the plasma membrane. It has distinct functions in regulating hepatitis, cirrhosis, and hepatocarcinogenesis. Given the increasing number of cases of liver cancer, nonalcoholic steatohepatitis, and non-alcoholic fatty liver disease worldwide, investigations on the role of Cav1 in liver diseases are warranted. In this study, we aimed to investigate the role of Cav1 in the pathogenesis of acute liver injury. Wild-type (WT) and Cav1 knockout (KO) mice (Cav1^tm1Mls^) were injected with carbon tetrachloride (CCl_4_). Cav1 KO mice showed significantly reduced degeneration, necrosis, and apoptosis of hepatocytes and decreased level of alanine transaminase (ALT) compared to WT mice. Moreover, Cav1 was required for the recruitment of hepatic macrophages. The analysis of the mRNA levels of CD86, tumor necrosis factor (TNF), and interleukin (IL)-6, as well as the protein expression of inducible nitric oxide synthase (iNOS), indicated that Cav1 deficiency inhibited the polarization of hepatic macrophages towards the M1 phenotype in the injured liver. Consistent with *in vivo* results, the expressions of CD86, TNF, IL-6, and iNOS were significantly downregulated in Cav1 KO macrophages. Also, fluorescence-activated cell sorting (FACS) analysis showed that the proportion of M1 macrophages was significantly decreased in the liver tissues obtained from Cav1 KO mice following CCl_4_ treatment. In summary, our results showed that Cav1 deficiency protected mice against CCl_4_-induced acute liver injury by regulating polarization of hepatic macrophages. We provided direct genetic evidence that Cav1 expressed in hepatic macrophages contributed to the pathogenesis of acute liver injury by regulating the polarization of hepatic macrophages towards the M1 phenotype. These findings suggest that Cav1 expressed in macrophages may represent a potential therapeutic target for acute liver injury.

## Introduction

Hepatic fibrosis is a wound-healing response of the liver against damage or insult, characterized by inflammatory responses. The inflammation and subsequent damage of the liver can be induced by a variety of stimuli, including chronic viral hepatitis B and C, autoimmune and biliary diseases, alcoholic steatohepatitis, and nonalcoholic steatohepatitis (1, 2). Liver macrophages, accounting for 20–35% of hepatic non-parenchymal cells, represent the largest proportion (80–90%) of tissue macrophages in the host. Resident and recruited macrophages are essential for the homeostasis of the liver and its response to tissue damage. Activated macrophages can be classified into ‘pro-inflammatory’ M1 and ‘immunoregulatory’ M2 macrophages, though this simple dichotomous nomenclature does not fully reflect the complex biology of macrophage subsets (3, 4). M1 macrophages, characterized by CD86 expression, are activated by interferon γ(IFN-γ), lipopolysaccharide (LPS), or high-mobility group protein 1. Activated M1 macrophages are pro-inflammatory cells that produce numerous inflammatory cytokines, including tumor necrosis factor (TNF)-α, interleukin (IL)-1, and IL-6 (5, 6). In contrast, M2 macrophages suppress inflammatory responses and facilitate tissue repair by upregulating IL-10, IL-4, and IL-13 (7, 8). Macrophage polarization plays an important role in inflammatory responses, during which it may protect against uncontrolled inflammation. Therefore, maintaining the balance between M1 and M2 macrophages is the key to treat various inflammatory disorders (9, 10).

Caveolin-1 (Cav1) is the structural protein of caveolae, the invaginations of the plasma membrane that function as transport carriers and signaling platforms on the membrane. Recent studies have reported that Cav1 plays a crucial role in liver function and the progression of hepatic diseases, including cholestasis, hepatitis, cirrhosis, and hepatocarcinogenesis (11–14). The upregulation of Cav1 has been observed in patients with cirrhosis and rats with bile duct ligation-induced cholestatic liver injury (15, 16). Cav1 deficiency has been shown to aggravate ConA-induced hepatocellular death and ferroptosis associated with excessive nitrogen stress (17). However, it has also been reported that Cav1 deletion reduces inflammatory responses. Cav1 deficiency suppressed nuclear factor κB (NF-κB) activation and alleviated lung injury in mice challenged with LPS (18). Cav1 knockout (KO) also alleviated acute liver injury by inhibiting TLR4/NF-κB-mediated inflammatory responses in mice (19). Therefore, the role of Cav1 in inflammatory responses may vary depending on stimuli.

Cav1 is involved in the activation and polarization of macrophages. A previous study showed that high-density lipoproteins (HDLs) inhibited the polarization of macrophages to an M1 phenotype by downregulating Cav1 on the membrane of M1 macrophages (20). Also, Cav1 deletion exacerbated cardiac interstitial fibrosis in mice by promoting M2 macrophage activation after myocardial infarction (21). In murine alveolar and peritoneal macrophages, Cav1 deficiency increased the production of pro-inflammatory cytokines (i.e. TNF-α and IL-6) following LPS stimulation, but downregulated anti-inflammatory cytokine IL-10 (22). Furthermore, cavtratin, a cell-permeable peptide of Cav1 inhibited the survival and migration of macrophages *via* targeting c-Jun N-terminal kinase (JNK) (23). The activation of Cav1 was involved in the autophagy of RAW 264.7 cells and bone marrow-derived macrophages (BMDMs), which increased the production of IL-10 in macrophages and therefore reduced inflammation in LPS-induced sepsis and liver injury (24).

In this study, we investigated the role of Cav1 in the polarization of hepatic macrophages in carbon tetrachloride (CCl_4_)-induced acute liver injury. Our results showed that Cav1 deficiency attenuated liver injury, as evidenced by reduced degeneration, necrosis, and apoptosis of hepatocytes and decreased expression of alanine transaminase (ALT). Furthermore, Cav1 KO inhibited macrophage infiltration and downregulated the expressions of CD86, TNF, IL-6, and inducible nitric oxide synthase (iNOS). Then, we isolated primary hepatic macrophages from wild-type (WT) and Cav1 KO mice. Consistent with *in vivo* data, the levels of pro-inflammation factors, including CD86, TNF, IL-6, and iNOS, were significantly decreased in macrophages obtained from Cav1 KO mice. Moreover, fluorescence-activated cell sorting (FACS) analysis showed that the proportion of M1 macrophages was significantly decreased in the liver tissues obtained from Cav1 KO mice following CCl_4_ treatment. Taken together, Cav1 is a key regulator in the activation and polarization of hepatic macrophages in acute liver injury.

## Materials and Methods

### Mouse Strain

Cav1 KO mice (Cav1^-/-^, STOCK Cav1^tm1Mls^/J) and WT mice (B6129SF2/J) were purchased from Jackson Laboratory. The experimental protocol was approved by the Animal Experiments and Experimental Animal Welfare Committee of Capital Medical University (Approval Number: AEEI-2016-150, 16, October, 2016). All animal studies were performed in accordance with the Guidelines of the Animal Experiments and Experimental Animals Management Committee of Capital Medical University.

### Mouse Model of Acute Liver Injury

Previous studies have indicated that female animals are more resistant to CCl_4_-induced liver injury than male animals (25, 26). Therefore, male Cav1 KO mice and WT mice (8-10 weeks old, weighing 25-28 g) were used in this study. A mouse model of acute liver injury was established by intraperitoneally injecting mice with a mixture of CCl_4_ and olive oil (1:9 v/v) at a dose of 10 µL/g body weight, twice per week. Control mice were injected with olive oil alone at 10 µL/g body weight, twice weekly. Mice were sacrificed at 1 day, 3 days, 1 week, 2 weeks, and 4 weeks after CCl_4_ treatment (n=6) by CO_2_ exposure. Liver tissues were collected for hematoxylin and eosin (H&E) staining, TUNEL staining, immunohistochemical staining, real-time PCR (RT-PCR), Western blot, ELISA, and flow cytometry (FCM) analyses. Serum samples were isolated for the measurement of the ALT level.

### H&E Staining

Liver tissues were fixed in 2% paraformaldehyde for histological examination. Paraffin-embedded liver tissues were cut into 4-μm-thick sections, deparaffinized, and stained with H&E. The necrotic areas of the liver were marked with black curves. All tissue sections were examined under an Olympus BH-2 microscope (Olympus Optical Co. Ltd., Beijing, China). Motic Images 2000 (Motic China Group Co. Ltd., Guangzhou, China) was used to measure the proportion of necrotic area relative to the entire visual field.

### TUNEL Staining

Liver tissues were embedded on dry ice, cut into 5-μm-thick sections, and fixed with 4% paraformaldehyde. TUNEL staining was performed to analyze hepatocyte apoptosis according to the manufacturer’s instruction (Roche Diagnostics GmbH, Mannheim, Germany). Liver sections were incubated with 50 μL of TUNEL reaction mixture for 60 min at 37°C. Samples were observed under a fluorescence microscope at a range of 515–565 nm (green).

### Measurement of Serum ALT Level

Blood was obtained from the orbital venous plexus of mice at 7 and 14 days after CCl_4_ treatment. The serum ALT level was determined using a standard enzymatic assay kit (Jiancheng Bioengineering Institute, Nanjing, China). The highly-colored end product was detected at 490–520 nm by a spectrophotometer (Hitachi 736–10, Beijing, China). The absorbance of the end product was proportional to the enzyme’s activity.

### Determination of Cytokines and NO

One mL of PBS was added to 0.5g liver tissues from mice and homogenized and centrifuged at 13,000 rpm for 10 min. According to the manufacturer’s instructions, the level of TNF-α (AMEKO, Shanghai, China), interleukin (IL)-6 (AMEKO, Shanghai, China), and NO (Beyotime Biotechnology, Shanghai, China) in the supernatants of the liver tissue homogenates were measured by ELISA.

### Immunohistochemical Staining

Immunohistochemical staining for F4/80 (Cat. No. 610406, 1:1000, Abcam, Shanghai, China) was performed using an immunohistochemistry kit (Boster Biological Engineering Co., Wuhan, China) according to the manufacturer’s instructions. The yellow-stained areas in the sections were semi-quantitatively analyzed by an image analyzer (Image-Pro Plus, MediaCybernetics, Rockville, MD, USA). The results were shown as area density (area of the positive cells/area of the whole field).

### RT-qPCR

Total RNA was extracted from liver tissues or hepatic macrophages by Total RNA Extraction Kit (GenePool, Cat. No. GPQ1801) according to the manufacturer’s instructions. Primers were designed as follows: Mouse GAPDH: sense, 5’-CGA GAA TGG GAA GCT TGT CA-3’; antisense, 5’-TTG GCT CCA CCC TTC AAG T-3’. Mouse CD86: sense, 5’-TCC AAG TTT TTG GGC AAT GTC-3’; antisense, 5’-CCT ATG AGT GTG CAC TGA GTT AAA CA-3’. Mouse TNF: sense, 5’-AAA GCA TGA TCC GCG ACG TG-3’; antisense, 5’-AGG AAT GAG AAG AGG CTG AGA CA-3’. Mouse IL-6: sense, 5’-TAG TCC TTC CTA CCC CAA TTT CC-3’; antisense, 5’-TTG GTC CTT AGC CAC TCC TTC-3’. Mouse iNOS: sense, 5’-ATG GTC CGC AAG AGA GTG CT-3’; antisense, 5’-TAA CGT TTC TGG CTC TTG AGC TG-3’. All real-time RT-PCR reactions were performed in an ABI Prism 7500 Sequence Detection System (Applied Biosystems, Foster City, CA, USA).

### Western Blot

Total protein (50 μg) extracted from liver tissues or hepatic macrophages was used for Western blot analysis. The following primary antibodies were used: rabbit anti-iNOS monoclonal antibody (1:1000, Abcam), rabbit anti-GAPDH monoclonal antibody (1:10000, Abcam) or Mouse anti-Actin monoclonal antibody (1:10000, Abcam). Goat anti-mouse IgG labeled with HRP (Cat. No. ab6789, 1:2000, Abcam) was used as the secondary antibody. Immunoreactive signals were detected using an Enhanced Chemiluminescence (ECL) kit (Amersham Pharmacia Biotech) through an ECL system. The results were quantified using Image J 1.43 (National Institutes of Health, Bethesda, MD) after densitometric scanning of the films. Western blot signals were normalized relative to the image of the appropriate control samples. Results of a minimum of three independent Western blot analyses were averaged and pooled to yield the data shown in the histograms.

### Isolation of Primary Hepatic Macrophages

Primary hepatic macrophages were isolated from Cav1 KO and WT mice as previously described (27). In brief, mice were anesthetized with an open midline incision and portal vein intubation. The liver was sequentially perfused with 0.04% collagenase IV (Sigma-Aldrich, Shanghai, China) in Dulbecco’s modified Eagle’s medium (Sigma-Aldrich, Shanghai, China) at a rate of 10 mL/min through the portal vein. Perfused livers were dissected and teased through 70-mm nylon mesh cell strainers (BD Biosciences, Breda, The Netherlands) and suspended in RPMI 1640 medium (Sigma-Aldrich, Shanghai, China). Then, a 25%/50% two-step percoll (Sigma-Aldrich, Shanghai, China) gradient was made and cell suspension was added to the top of 25% percoll. After centrifugation at 1500 g for 15 min, primary hepatic macrophages were collected from the top of 50% percoll and cultured in RPMI 1640 medium supplemented with 10% fetal bovine serum and antibiotics. Then cells were collected for RT-PCR and Western blot analysis.

### FCM

Primary hepatic macrophages isolated from Cav1 KO and WT mice were resuspended in ice-cold 10% FBS-PBS after density gradient centrifugation to a concentration of 1×10^6^ per milliliter. Then the antibody against FITC-F4/80 (ab60343), APC-CD86 (ab218757), FITC-IgG2a (ab18446), or APC-IgG2b (ab154434) were added to cell suspension at recommended concentrations. FITC-IgG2a and APC-IgG2b were used as the isotype control. After 30 min of incubation in the dark at 4°C, cells were washed three times with ice-cold 10% FBS-PBS and subjected to FCM analysis. FCM was performed on BD LSRFortessa and analyzed using BD FACSDiva 7.0 and Flowjo-V10.

### Statistical Analysis

Data were presented as mean ± standard deviation (SD). Statistical differences between groups were analyzed by one-way analysis of variance and subsequent Bonferroni *post hoc* test using the SPSS 17.0 software (SPSS Institute, Chicago, IL, USA). The *p*-value was two-tailed and considered statistically significant or highly significant when *p*<0.05 or *p*<0.01, respectively.

## Results

### Cav1 Deficiency Reduced Degeneration, Necrosis, and Apoptosis of Hepatocytes

**Figure 1:**
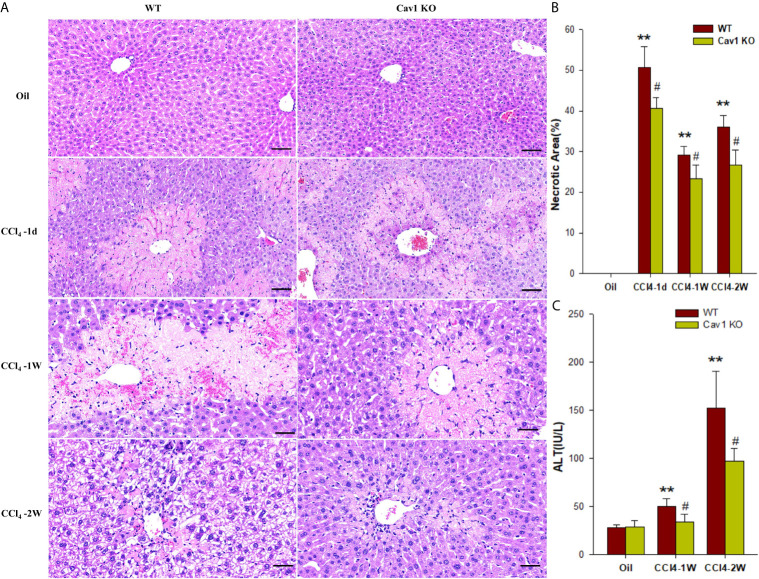
Cav1 deficiency attenuated degeneration and necrosis of hepatocytes in CCl_4_-induced liver damage. Cav1 KO and WT mice were intraperitoneally injected with CCl_4_ (10 μL/g of body weight) or olive oil. Liver tissues and serum samples were collected at 1 day, 3 days, 1week, and 2 weeks after injection. **(A)** Representative images of H&E-stained liver sections. Degeneration and necrosis of hepatocytes were observed around the central vein. **(B)** Quantitative analysis of hepatocytes necrosis. Cav1 KO mice showed significantly reduced necrotic area in the liver from 1-day to 2-week post-injection compared to the WT group. **(C)** The serum concentration of ALT was also significantly decreased in Cav1 KO mice. Data are shown as mean ± SD, **p < 0.01 compared with the WT+oil group, ^#^ *p* < 0.05, compared with the WT+CCl_4_ group at the same time point. Scale bar = 50 µm. n=6 per group.

Our previous study showed that the protein expression of Cav1 was significantly decreased in the injured livers of mice from 3–28 days after CCl_4_ treatment (28). To explore how Cav1 regulated inflammatory responses in acute liver injury, we analyzed the degeneration and necrosis of hepatocytes in Cav1 KO and WT mice using H&E staining (**Figure 1A**). WT and Cav1 KO mice treated with oil showed no spontaneous inflammation. The degeneration and necrosis of hepatocytes were observed around the central vein of the liver of both WT and Cav1 KO mice one day after CCl_4_ treatment. Normal hepatocytes were also found around the central vein of Cav1 KO mice, but not in the WT group. At 1 day, 3 days, and 2 weeks after CCl_4_ treatment, the necrotic area in Cav1 KO mice was significantly reduced compared to the WT controls (**Figure 1B**: 50.73% *vs.* 40.69, 29.12% *vs.* 23.28%, 36.03% *vs.* 26.63, respectively). We further measured the serum level of ALT to indicate liver damage. The ALT level was increased at 1 week and 2 weeks after CCl_4_ injection in WT mice. However, these changes were significantly diminished in Cav1 KO mice (**Figure 1C**: 50.00 IU/L *vs.* 34.00 IU/L, 152.60 IU/L *vs.* 97.00 IU/L, respectively), indicating that Cav1 deletion attenuated liver damage *in vivo*.

**Figure 2:**
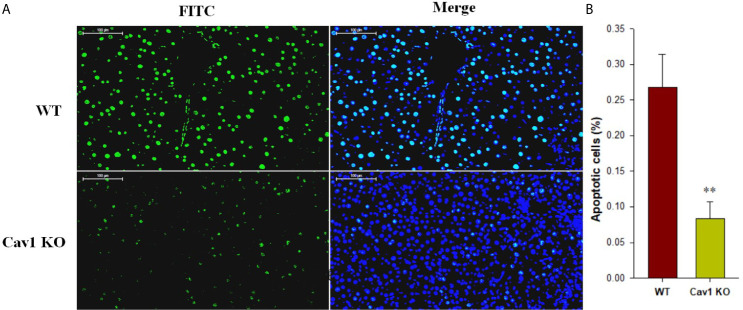
Cav1 deficiency reduced hepatocellular apoptosis in CCl_4_-induced acute liver injury. **(A)** The effect of Cav1 deficiency on CCl_4_-induced apoptosis in the liver was explored by TUNEL staining. Nuclei were counterstained with DAPI (blue) and apoptotic cells were detected by TUNEL (green). **(B)** Quantitative analysis of apoptosis in injured livers. Significantly decreased apoptosis was observed in the liver of Cav1 KO mice compared to the WT group at 2 weeks after CCl_4_ treatment. Data are shown as mean ± SD, **p < 0.01 compared with WT mice. Scale bar = 100 µm. n=6 per group.

Hepatocyte apoptosis contributes the most to liver damage in acute liver injury. The apoptosis of hepatocytes following CCl_4_ injection was measured by TUNEL staining (**Figure 2A**). The WT group showed a large proportion of TUNEL‐positive hepatocytes (27%) at 2 weeks after CCl_4_ administration. In Cav1 KO mice, however, the percentage of TUNEL-positive hepatocytes was significantly reduced (8%) compared with the WT controls (**Figure 2B**). Collectively, these results suggested that the absence of Cav1 alleviated CCl_4_-induced liver injury *in vivo*.

### Cav1 Deficiency Inhibited Infiltration and Polarization of Hepatic Macrophages

**Figure 3:**
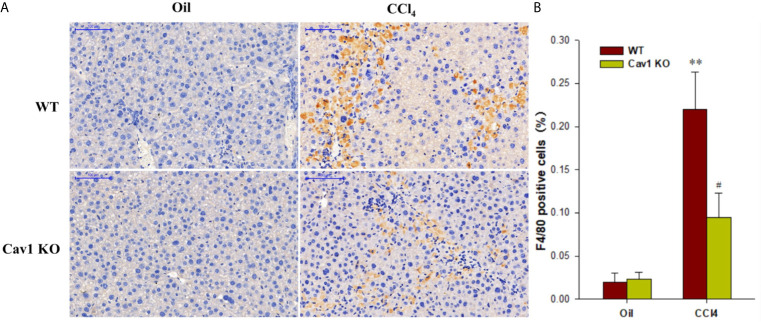
Infiltration of macrophages was reduced in Cav1 KO mice. Liver tissues were collected from Cav1 KO and WT mice after CCl_4_ treatment and stained for F4/80 for 24 h by immunohistochemistry. **(A)** Representative immunohistochemical staining for F4/80 (yellow). F4/80 antigen is a murine macrophage-specific antigen. Nuclei were counterstained with hematoxylin (blue). **(B)** The percentage of F4/80 positive cells relative to all cells was measured. Significantly decreased infiltration of macrophages was observed in Cav1 KO mice compared to the WT group at 2 weeks after CCl_4_ treatment. Data are shown as mean ± SD, **p < 0.01 compared with the WT+oil group, ^#^ *p* < 0.05, compared with the WT+CCl_4_ group. Scale bar = 100 µm. n=6 per group.

Hepatic macrophages play a central role in the pathogenesis of liver injury and have been proposed as potential targets for treating fibrosis (29). However, the role of Cav1 in macrophage activation remains unclear. Immunohistochemical staining (**Figure 3A**) showed that F4/80-positive macrophages accounted for 22% of all cells in the liver of WT mice at 1 week after CCl_4_ injection. However, the percentage of F4/80-positive macrophages in Cav1 KO mice was significantly reduced to 8% (**Figure 3B**). These data implied that Cav1 deficiency markedly reduced the infiltration of macrophages to the liver following CCl_4_ treatment.

**Figure 4:**
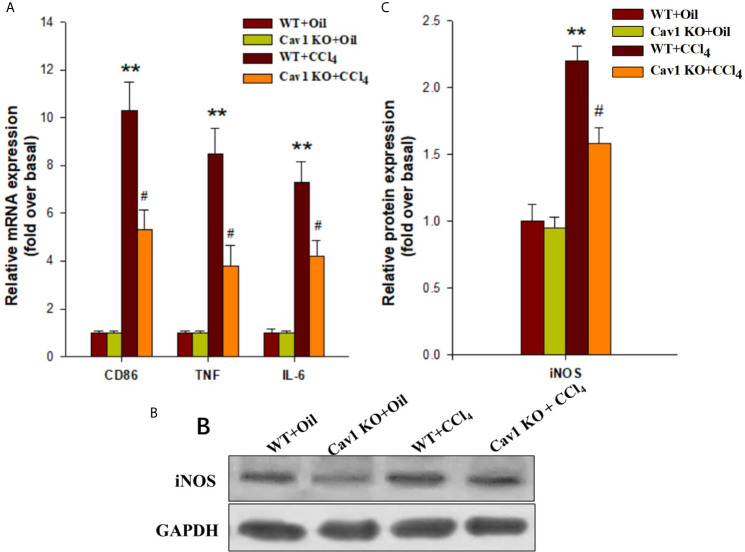
Cav1 deficiency reduced polarization of macrophages to the M1 phenotype in injured liver. The expression of M1 markers was measured by RT-qPCR and Western blot. **(A)** At two weeks after CCl_4_ administration, the mRNA expressions of M1 markers (i.e. CD86, TNF, and IL-6) were detected. **(B)** The expression of inducible nitric oxide synthase (iNOS) was measured by Western blot. **(C)** Quantitative analysis of iNOS expression in the injured liver. M1 markers were significantly downregulated in the liver of Cav1 KO mice compared to the WT group at 2 weeks after CCl_4_ treatment. Data are shown as mean ± SD, **p < 0.01 compared with the WT+oil group, ^#^ *p* < 0.05 compared with the WT+CCl_4_ group. n=6 per group.

Next, we investigated the effects of Cav1 on the activation and polarization of macrophages during acute liver injury. The mRNA expression of CD86 (M1 macrophage marker) in the liver of WT mice was significantly upregulated at 2 weeks after CCl_4_ administration (10-fold increase). We also measured the expression of M1 signature genes (i.e. TNF and IL-6) in the injured liver. The mRNA levels of TNF and IL-6 in the liver of CCl_4_-treated WT mice were also increased at 2 weeks after treatment (8-fold and 7-fold increase, respectively) compared to the WT+Oil group. Moreover, the mRNA levels of CD86, TNF, and IL-6 in the liver of Cav1 KO mice were significantly reduced (by ~50%) compared to the WT controls (**Figure 4A**). We then measured the protein expression of iNOS (M1 macrophage marker, **Figure 4B**) in different groups of mice. Consistent with the results of mRNA expression analysis, Cav1 deficiency significantly decreased the protein level of iNOS after CCl_4_ injection (**Figure 4C**). Furthermore, Cav1 KO also significantly reduced TNF-α, IL-6 (**Figure S1**) and NO production (**Figure S2**) in mice livers induced by CCl4. These findings demonstrated that Cav1 KO inhibited the activation of macrophages and their polarization to the M1 phenotype in the injured liver, suggesting that Cav1 may act as a pro-inflammatory factor in CCl_4_-induced liver injury.

### Cav1 Was Critical for the Activation and Polarization of Hepatic Macrophages

**Figure 5:**
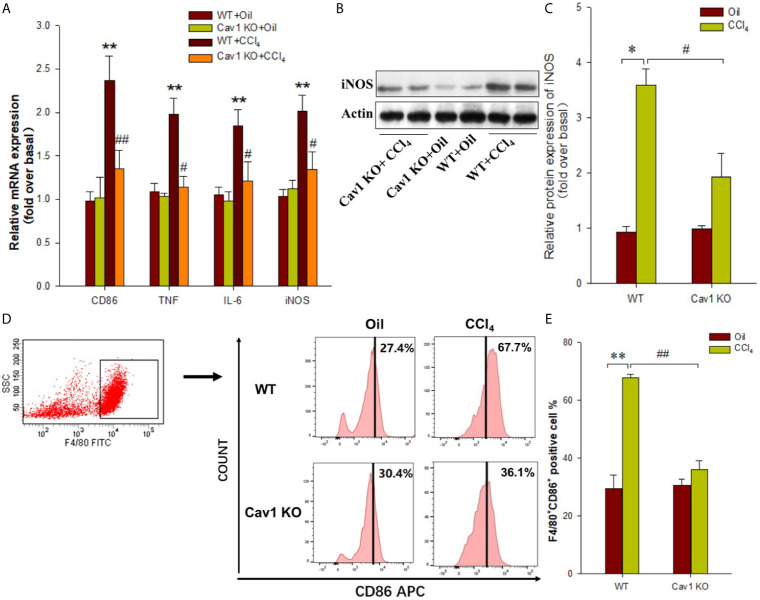
Cav1 deficiency reduced hepatic macrophages polarization toward M1 phenotype in injured Liver. Mouse primary hepatic macrophages were isolated from Cav1 KO and WT mice treated with olive oil or CCl_4_ for one week. **(A)** The mRNA levels of M1 markers (i.e. CD86, TNF, IL-6, and iNOS) were detected by RT-PCR. **(B)** The protein expressions of iNOS (M1 marker) were measured by Western blot. **(C)** Histogram showing densitometry analysis and quantification of iNOS. **(D) **FCM was performed to detect M1 macrophages. **(E)** Quantification of the proportion of M1 macrophages. Data are shown as mean ± SD, *p < 0.05, **p < 0.01 compared with the WT+oil group; ^#^ *p* < 0.05, ^##^ *p* < 0.01 compared with the WT+CCl_4_ group. n=6 per group.

To confirm the role of Cav1 in the activation and polarization of macrophages *in vivo*, we isolated hepatic macrophages from WT and Cav1 KO mice treated with CCl_4_ for one week. The expression of M1 markers in macrophages was analyzed by RT-PCR and Western Blot. The mRNA levels of CD86, TNF, IL-6, and iNOS in macrophages were increased after CCl_4_ treatment, indicating polarization of macrophages towards the M1 phenotype. Cav1 KO, however, significantly suppressed CCl_4_-induced upregulation of M1 signature genes in isolated macrophages (**Figure 5A**). Furthermore, CCl_4_ administration upregulated the protein expression of iNOS in macrophages at 2 weeks after treatment (2-fold increase) compared to the WT+Oil group, but this effect was abolished by Cav1 deficiency (~50% reduction) (**Figure 5B, C**).

Hepatic macrophages were isolated from Cav1 KO and WT mice and sorted by F4/80^+^ gating. Gating procedure was shown in **Supplementary Material** (**Figure S3**). FACS analysis showed no significant difference in the proportion of M1 macrophages between WT and Cav1 KO mice treated with oil. After CCl_4_ treatment, the proportion of M1 macrophages in the liver of WT mice was significantly increased (WT+Oil *vs.* WT+CCl_4_: 27.4% *vs.* 67.7%). However, Cav1 KO suppressed CCl_4_-induced increase in the proportion of M1 macrophages (Cav1 KO+Oil *vs.* Cav1 KO+CCl_4_: 30.4% *vs.* 36.1%). Compared with WT mice, the proportion of F4/80^+^CD86^+^ hepatic macrophages was markedly reduced in Cav1 KO mice following CCl_4_ treatment (WT+CCl_4_ *vs.* Cav1 KO+CCl_4_: 67.7% *vs.* 36.1%) (**Figure 5D, E**). Altogether, these results suggested that Cav1 deficiency inhibited the polarization of macrophages towards the M1 phenotype during liver injury.

## Discussion

Cav1 is a fatty acid- and cholesterol-binding protein that constitutes the major structural component of caveolae. It is implicated in a variety of biological processes, such as endocytosis, transcytosis, signal transduction, and lipid metabolism. Recent evidence shows that Cav1 is an important regulator of liver function and diseases (14, 30, 31). In this study, we found that Cav1 KO attenuated necrosis and apoptosis of hepatocytes, and decreased serum ALT level in mice subjected to CCl_4_ treatment. Our findings were in line with a previous study, showing that alcohol-induced liver lesions were negatively correlated with the serum Cav1 level of binge drinkers and Cav1 protected hepatocytes from ethanol-mediated apoptosis by inhibiting iNOS activity and regulating the EGFR- and STAT3-signaling cascades (14). Furthermore, the downregulation of Cav1 in liver tissues contributed to ConA-induced hepatic damage (17). Cav1 was also reported to inhibit nitrative stress-induced liver damage during hepatic ischemia-reperfusion injury (32). In our previous report (28), increased ALT/AST levels were observed at 3 days after CCl_4_ injection. However, we observed a decrease in ALT levels in the Cav1-KO mice compared to WT controls 7 and 14 days after CCl_4_ treatment in this report. The dose of CCl_4_ and time points maybe the reasons why ALT results are different. It is possible that Cav1 may also has different roles depending on the stage of the liver disease as previously findings in lung injury (22, 33).

Although the molecular pathways that activate macrophages vary among different types of injuries, hepatic macrophages show a common response to liver injury. In general, Kupffer cells are early sensors of tissue damage, which respond to liver stress and may either stimulate or suppress immune responses (34, 35). Previous studies have suggested that Kupffer cells play an important role in liver injury (36, 37). They are professional phagocytes that sense tissue damage and subsequently activate pro-inflammatory cascades (38). Our previous study showed that the hepatic expression of Cav1 was decreased in CCl_4_-injected mice (28). Therefore, we assumed that Cav1 may regulate inflammatory responses in the liver by mediating macrophage recruitment. The effect of Cav1 on macrophage recruitment has been highlighted in previous studies (12, 39). Here, we examined whether Cav1 KO can affect the number of host cells recruited to the damaged site of the liver. There are two types of macrophages in damaged livers, the tissue-resident macrophages, Kupffer cells, and the infiltrated macrophages, BMMs (bone marrow-derived monocytes/macrophages). Resident macrophages have distinct characteristics, such as the requirement of different specific transcription factors and the expression of different markers. As previously reported, the expression of F4/80 is brighter in resident macrophages than in monocyte-derived macrophages (40). Therefore, the stronger labeling of F4/80 in WT comparing to Cav1 KO mice may suggest that mice deficient in Cav1 have less resident macrophages than WT.

During liver fibrogenesis, the number of macrophages was significantly increased in the liver; yet, the polarization of these cells was still unclear (41). Following the changes in the microenvironment, macrophages are polarized into two phenotypes, classically activated (M1) macrophages and alternatively activated (M2) macrophages (42). It has been found that M1 cells are pro-inflammatory macrophages and M2 are anti-inflammatory macrophages (43). M1 cells play an important role in the initial phase of diseases by releasing large amounts of factors. In contrast, M2 cells exert an anti-inflammatory effect by inducing the production of anti-inflammatory cytokines, such as IL-4 and IL-10 (44, 45). A previous study has suggested that M1, but not M2, macrophages play critical roles in CCl_4_-induced liver injury (46). Cav1 is a key regulator of macrophage polarization. It has been confirmed that Cav1 is required for HDLs to inhibit M1 induction as BMDMs from Cav1 KO mice continued to polarize into the M1 phenotype despite the presence of HDLs (20). Moreover, Cav1 deletion promotes a multitude of maladaptive repair processes after myocardial infarction, including increased M2 macrophage infiltration and dysregulated M1/M2 balance (21). M1 macrophages are highly involved in CCl_4_-induced liver injury in mice (46). In this study, we investigated the effect of Cav1 on macrophage activation and polarization. Our results showed that the expressions of M1 signature genes (i.e. CD86, TNF, IL-6, and iNOS) were significantly reduced in the liver of Cav1 KO mice compared with the WT group. We further isolated hepatic macrophages from Cav1 KO and WT mice following CCl_4_ injection. The expressions of M1 phenotype markers, including CD86, TNF, IL-6, and iNOS, were downregulated in Cav1 KO macrophages, indicating that the proportion of M1-polarized macrophages was significantly reduced in the absence of Cav1. We also examined the effect of Cav1 KO on the polarization of hepatic macrophages. The proportion of F4/80^+^CD86^+^ macrophages in isolated primary mouse hepatic macrophages was measured. Compared with the WT controls, F4/80^+^CD86^+^ macrophages were markedly reduced in the Cav1 KO group. Both *in vivo* and *in vitro* data suggested Cav1 was required for the polarization of macrophages towards the M1 phenotype during liver injury. Further investigations are needed to clarify the effects of Cav1 on the polarization of Kupffer cells and BMDMs *in vivo*. The clodronate liposome can be used to remove Kupffer cells and the CCl_4_ treatment can be used to induce liver injury in mice pretreated with or without small interfering RNAs targeting Cav1.

In summary, our results showed that Cav1 deficiency markedly suppressed CCl_4_-induced necrosis and apoptosis of hepatocytes in mice. The protective effect of Cav1 KO was associated with reduced macrophage infiltration and polarization to the M1 phenotype. This study provided direct genetic evidence that Cav1 expressed in hepatic macrophages contributed to the pathogenesis of acute liver injury by regulating macrophage polarization towards the M1 phenotype. Even though only male animals were used in this study, we expect Cav1 deficiency also alleviated acute liver injury in female. Cav1 represents a promising therapeutic target for acute liver injury.

## Data Availability Statement

The raw data supporting the conclusions of this article will be made available by the authors, without undue reservation.

## Ethics Statement

The animal study was reviewed and approved by Animal Experiments and Experimental Animals Management Committee of Capital Medical University.

## Author Contributions

ZY, JZ, YW, acquisition of data, analysis and interpretation of data. JL, QS, study design, financial support, drafting of the manuscript. All authors contributed to the article and approved the submitted version.

## Funding

This work was supported by the grant from the National Natural and Science Foundation of China (81600481).

## Supplementary Material

The Supplementary Material for this article can be found online at: https://www.frontiersin.org/articles/10.3389/fimmu.2021.713808/full#supplementary-material

## Conflict of Interest

The authors declare that the research was conducted in the absence of any commercial or financial relationships that could be construed as a potential conflict of interest.

## Publisher’s Note

All claims expressed in this article are solely those of the authors and do not necessarily represent those of their affiliated organizations, or those of the publisher, the editors and the reviewers. Any product that may be evaluated in this article, or claim that may be made by its manufacturer, is not guaranteed or endorsed by the publisher.
