# Supplementary material for: Caveolin-1 Deficiency Protects Mice Against Carbon Tetrachloride-Induced Acute Liver Injury Through Regulating Polarization of Hepatic Macrophages
Source: Front Immunol. 2021 Aug 9;12:713808. doi: 10.3389/fimmu.2021.713808 (PMC8380772; doi:10.3389/fimmu.2021.713808)
Supplement: Supplementary file 1 [file DataSheet_1.docx]

**Supplements**

**Figure 1 Cav1 deficiency reduced TNF-α and IL-6 production in mouse liver**

**
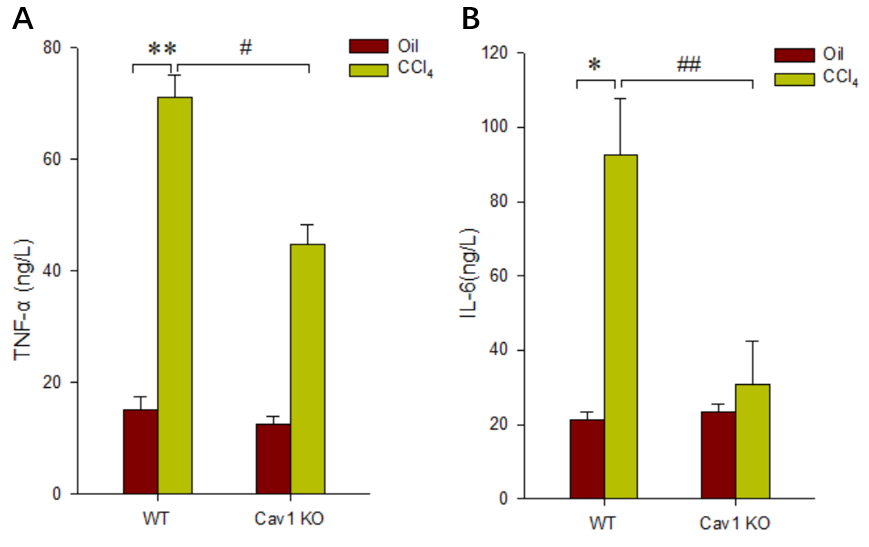
**

Liver tissues were collected from Cav1 KO and WT mice after CCl_4_ treatment for 24 h.

One mL of PBS was added to 0.5g liver tissues from mice and homogenized and centrifuged at 13,000 rpm for 10 min. The level of TNF-α (A) and interleukin (IL)-6 (B)in the supernatants of the liver tissue homogenates was measured by ELISA. TNF-α and IL-6 were significantly downregulated in the liver of Cav1 KO mice compared to the WT group. Data are shown as mean±SD, **p*<0.05, ***p*<0.01 compared with the WT+oil group, #*p*<0.05, ##*p*<0.01 compared with the WT+CCl_4_ group. n=6 per group.

**Figure 2 Reduced production of NO in Cav1 KO mice**


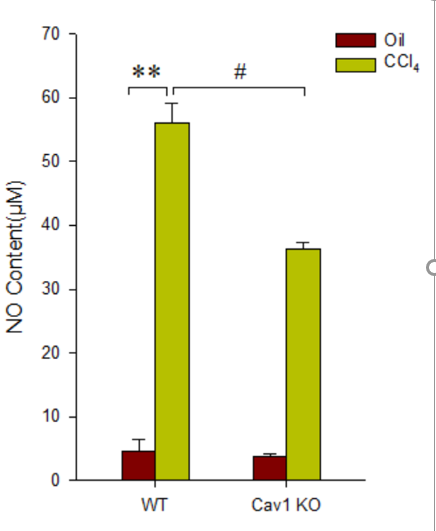


Liver tissues were collected from Cav1 KO and WT mice after CCl_4_ treatment for 24 h.

One mL of PBS was added to 0.5g liver tissues from mice and homogenized and centrifuged at 13,000 rpm for 10 min. The content of NO in the supernatants of the liver tissue homogenates was measured by ELISA. NO was significantly decreased in the liver of Cav1 KO mice compared to the WT group. Data are shown as mean±SD, ***p*<0.01 compared with the WT+oil group, *#*p<0.05 compared with the WT+CCl_4_ group. n=6 per group.

**Figure 3 FSC x SSC and FSC-H x FSC-A gating before F4/80 and CD86 analyzing.**


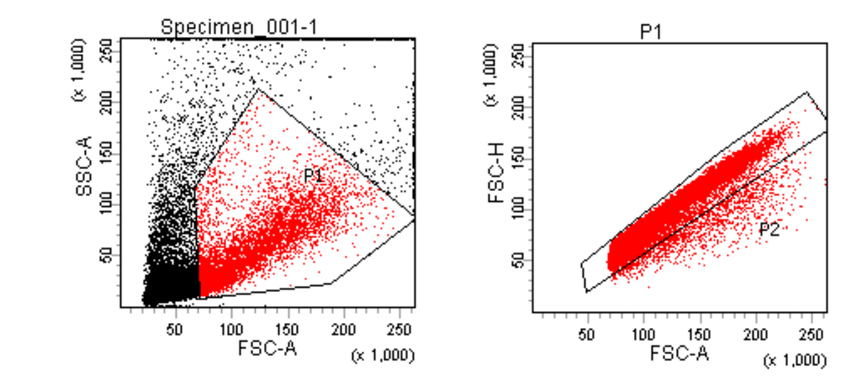


Mouse primary hepatic macrophages were isolated from Cav1 KO and WT mice treated with olive oil or CCl4 for one week. By using FCS x SSC first to gate the region of monocytes group P1, then FSC-H x FSC-A was used to gate P2 (the separated cells) before analyzed with M1 macrophages markers F4/80 and CD86.
